# A novel visual facial anxiety scale for assessing preoperative anxiety

**DOI:** 10.1371/journal.pone.0171233

**Published:** 2017-02-14

**Authors:** Xuezhao Cao, Roya Yumul, Ofelia Loani Elvir Lazo, Jeremy Friedman, Omar Durra, Xiao Zhang, Paul F. White

**Affiliations:** 1 Department of Anesthesiology, Cedars-Sinai Medical Center, Los Angeles, California, United States of America; 2 Department of Anesthesiology, The First Hospital of China Medical University, Shenyang, Liaoning, China; 3 Department of Anesthesiology, David Geffen School of Medicine-UCLA, Los Angeles, California, United States of America; 4 Department of Anesthesiology, White Mountain Institute, The Sea Ranch, California, United States of America; Radboud Universiteit, NETHERLANDS

## Abstract

**Background:**

There is currently no widely accepted instrument for measuring preoperative anxiety. The objective of this study was to develop a simple visual facial anxiety scale (VFAS) for assessing acute preoperative anxiety.

**Methods:**

The initial VFAS was comprised of 11 similarly styled stick-figure reflecting different types of facial expressions (Fig 1). After obtaining IRB approval, a total of 265 participant-healthcare providers (e.g., anesthesiologists, anesthesiology residents, and perioperative nurses) were recruited to participate in this study. The participants were asked to: (1) rank the 11 faces from 0–10 (0 = no anxiety, while 10 = highest anxiety) and then to (2) match one of the 11 facial expression with a numeric verbal rating scale (NVRS) (0 = no anxiety and 10 = highest level of anxiety) and a specific categorical level of anxiety, namely no anxiety, mild, mild-moderate, moderate, moderate-high or highest anxiety. Based on these data, the Spearman correlation and frequencies of the 11 faces in relation to the 11-point numerical anxiety scale and 6 categorical anxiety levels were calculated. The highest frequency of a face assigned to a level of the numerical anxiety scale resulted in a finalized order of faces corresponding to the 11-point numeric rating scale.

**Results:**

The highest frequency for each of the NVRS anxiety scores were as follow: A0, A1, A2, A3, A4, A5, A7, A6, A8, A9 and A10 (Fig 2). For the six categorical anxiety levels, a total of 260 (98.1%) participants chose the face A0 as representing ‘no’ anxiety, 250 (94.3%) participants chose the face A10 as representing ‘highest’ anxiety and 147 (55.5%) participants chose the face A8 as representing ‘moderate-high’ anxiety. Spearman analysis showed a significant correlation between the faces A3 and A5 assigned to the mild-moderate anxiety category (r = 0.58), but A5 was ultimately chosen due to its higher frequency compared to the frequency of A3 (30.6% vs 24.9%)(Fig 3). Similarly, the correlation of the faces A7 and A6 was significantly correlated with moderate anxiety (r = 0.87), but A7 remained because of its higher frequency (35.9% vs 22.6%). Using frequency and Spearman correlations, the final order of the faces assigned to the categories none, mild, mild-moderate, moderate, moderate-high and highest anxiety levels was A0, A1, A5, A7, A8 and A10, respectively (Fig 4).

**Conclusion:**

The proposed VFAS was a valid tool for assessing the severity of acute [state] anxiety, and could be easy to administer in routine clinical practice.

## Introduction

Up to 80% of patients experience acute preoperative anxiety prior to elective surgery, and can have both physiologic and psychological consequences [1]. Previous studies have demonstrated that excessive preoperative anxiety increases the intraoperative anesthetic requirement and can prolong recovery [2]. A rapid and objective assessment of preoperative anxiety might be useful for improving perioperative patient care and potentially enhancing the recovery process.

The current “gold standard” for evaluating acute anxiety is the State-Trait Anxiety Inventory (STAI) which consists of two separate 20-item questionnaires [3]. The Brief Symptom Inventory has 53 items which measures several affective states including anxiety [4]. However, these anxiety measurement tools are time consuming which limit their usefulness in the preoperative setting. The numeric verbal rating scale (NVRS) has been utilized for assessing preoperative anxiety. However, this 11-point scale requires that a patient describe their acute anxiety level in numerical terms [5]. Furthermore, there is a poor correlation (coefficient = 0.50) between the NVRS and the Spielberger State Anxiety Inventory scores [6]. Ekman and colleagues have shown that facial muscle patterns can be used to reliably detect emotions and to distinguish between differing types of emotions (e.g., anger vs. fear) [7]. A facial affective scale has been previously validated as a reliable tool for assessing acute pain in children [8]. Additionally, a visual analog scale (VAS) is a simple self-rating tool for rapidly assessing the level of state anxiety [9]. Therefore, we speculated that a pictorial facial scale would be a suitable alternative to the simple NVRS and the VAS for assessing the patient’s level of state anxiety in the preoperative period. The objective of this preliminary investigation was to develop a user-friendly tool for the rapid assessment of state anxiety during the perioperative setting. The proposed visual facial anxiety scale (VFAS) was designed to allow surgical patients to identify their level of acute preoperative anxiety using simple facial depictions.

## Methods

**Fig 1:**
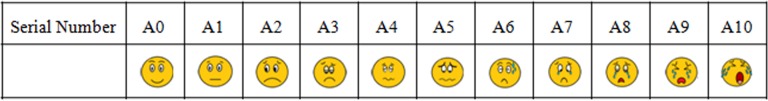
A total of 11 facial expressions were designed to reflect differing levels of stressfulness.

The research was approved by IRB of Cedars Sinai Medical Center (No: Pro00041348). The participants provide their written informed consent to participate in this study and the ethics IRBs approved this consent procedure. A total of 265 healthcare providers [anesthesiologists (n = 98), anesthesiology residents (n = 27) and perioperative nurses (n = 140)] at Cedars-Sinai Medical Center in Los Angeles, CA were invited to participate in this pilot study. The VFAS was comprised of 11 similarly styled facial expressions labeled A0 through A10 (the number was ‘blinded’ to the participants)(Fig 1).The NVRS were listed in order on one piece of paper, and the categorical anxiety levels, namely none, mild, mild-moderate, moderate, moderate-high and highest, were listed on a separate piece of paper. The participants were asked to: 1) match each separated face to a corresponding number, 0 (no anxiety) through 10 (highest anxiety), and 2) assign one face to each of six anxiety categorical anxiety variables: none, mild, mild-moderate, moderate, moderate-high and highest anxiety. The faces were purposely presented randomly to avoid any visual bias when assigning the faces to a number and a category.

### Statistical analysis

The frequency that a particular face was assigned to a number on the numerical scale and to an anxiety level on anxiety category (none, mild, mild-moderate, moderate, moderate-high and highest was determined. The Spearmen correlations were calculated to investigate the relationships of the VFAS to the six categorical anxiety levels. The highest frequency of a face assigned to a level of the numerical anxiety scale resulted in a finalized order of faces corresponding to the 11-point numeric rating scale.

## Results

**Fig 2:**
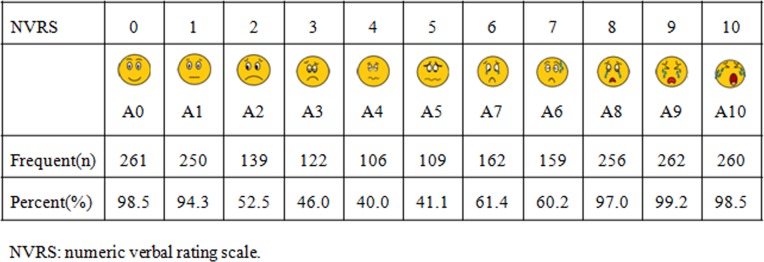
The highest frequencies of the 11 facial expressions.

Fig 2 shows which face had the highest frequency for each of the NVRS anxiety scores (0–10). A total of 261(98.5%) participants chose the face A0 as representing a 0 anxiety score, and 250 (94.3%) participants chose the face A1 as representing a score of 1. Similarly, more than 96% of participants chose the faces A8, A9 and A10 to represent anxiety scores of 8, 9 and 10, respectively (Fig 2). However, the results demonstrated obvious disagreement from levels 3–5. For example, the faces A1, A2, A3, A4, and A5 were all selected to represent anxiety score of 4, but A4 was ultimately chosen to represent anxiety level 4 because it was chosen with the greatest frequency. Finally, the order of the 11 faces best corresponding to the NVRS scores of 0–10 was determined to be A0, A1, A2, A3, A4, A5, A7, A6, A8, A9 and A10, respectively (Fig 2).

**Fig 3:**
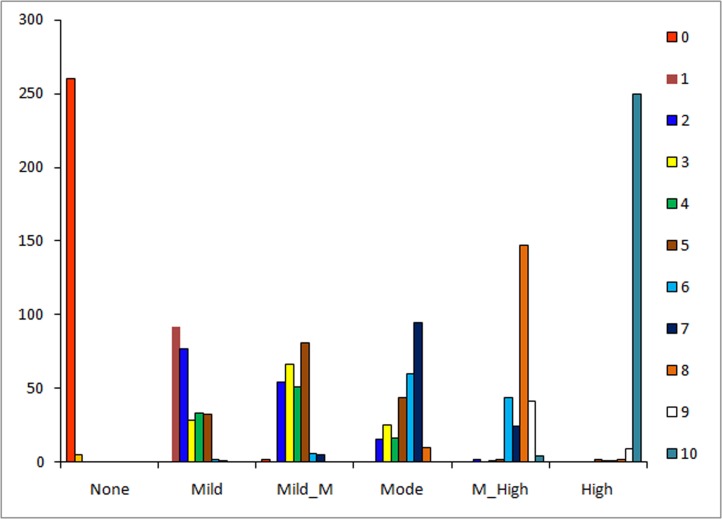
The frequency of 11 facial expressions in six anxiety categories.

**Fig 4:**
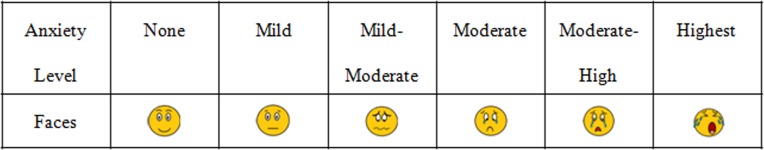
The proposed Visual Facial Anxiety Scale (VFAS).

For the association between the VFAS and the six categorical anxiety levels, 260 (98.1%) participants considered the face A0 as representing ‘no’ anxiety, 250 (94.3%) participants picked the face A10 as representing ‘highest’ anxiety and 147 (55.5%) participants chose the face A8 as representing ‘moderate-high’ anxiety (Fig 3). Predictably, there was a less obvious pattern for the other anxiety categories. Only 92 (34.7%) participants categorized the face A1 as depicting a mild level of anxiety, 81 (30.6%) participants categorized the face A5 into the mild-moderate anxiety level, and 95 (35.9%) considered the face A7 as representing a moderate level of anxiety (Fig 3). Further analysis using Spearman correlation determined which face would represent these three anxiety categories. Spearman analysis shows a significant correlation between the faces A3 and A5, which had the two highest frequencies assigned to the mild-moderate anxiety category (r = 0.58). A5 was ultimately chosen to represent the mild-moderate category of anxiety due to its higher frequency (30.6% vs 24.9%). A significant correlation was also found between the faces A7 and A6 in the moderate category (r = 0.87). The face A7 was selected to represent the moderate anxiety level because of its higher frequency (35.9% vs22.6%). Finally, the faces A0, A1, A5, A7, A8 and A10 were assigned to the six categories, none, mild, mild-moderate, moderate, moderate-high and highest anxiety levels, respectively (Fig 4).

## Discussion

Numerous facial scales have been used to measure pain intensity, including the Color Analog Scale [10], Face Pain Scale [11] and the Wong-Baker Face Pain Rating Scale [12]. Facial pain scales are validated and reliable tools for measuring acute pain [13]. A facial scale providing expression of emotions such as happiness, anger and anxiety is sensitive, reliable and easy to be understood [14]. Excessive anxiety prior to surgery can produce harmful physiological consequences [2].The measurement of preoperative anxiety could be useful in administering appropriate preoperative medication (e.g., an anxiolytic drug) to facilitate the induction and recovery process. The proposed VFAS is a simple six-point scale consisting of six faces representing an increasing level of anxiety from none (a neutral facial expression) to highest (a facial expression displaying extreme fear). In contrast to a NVRS which requires patients to transform their subjective anxiety state into numbers on a continuous scale from 0 to 10, the new VFAS as an instrument which utilizes recognizable facial representations of various anxiety states, thereby strengthening its construct validity. The correlation between the subject’s responses to the State-Anxiety Inventory questionnaire and to a Faces scale was found to be 0.70, a level regarded as providing evidence of criterion validity by experts in scale development and validation [15].

The facial scale is presented to patients as an alternative to a numeric linear analog scale (e.g., NVRS). Since it is often difficult for patients to quantify their level of anxiety using a simple numeric scale anchored by two extremes of anxiety (namely, no anxiety and highest level of anxiety), we proposed that a pictorial presentation of different facial expressions as a relatable measure of the patient’s levels of acute [state] anxiety. The healthcare provider merely has to ask the patient to choose which of the six facial expressions which most accurately reflects their level of anxiety at that precise moment in time. The new VFAS is quick and easy to administer, and therefore easy to incorporate into routine clinical practice.

In our previous pilot study, the facial scale was created using different style faces, and it was utilized as an alternative to the NVRS for assessing the level of preoperative [state] anxiety [16]. However, there were too many faces in this early scale and only a moderate correlation was found between the previous facial scale and NVRS. Furthermore, the inconsistent style of the 11 faces could result in confusion for the patient. The new six-facial VFAS was designed, and through analysis of frequency selection and Spearman correlation, the survey of the scale by anesthesiology and perioperative nursing personnel resulted in six faces being chosen to compose the new VFAS. In the testing of the order of the scale items, all participants independently placed the scale items in the order from the lowest to the highest anxiety level on an 11-point NVRS and then made associations with levels of acute anxiety on a six-point categorical rating scale. The highest frequency of a face assigned to a number on the NVRS resulted in a finalized order of faces. When the Spearman correlation between multiple faces and anxiety levels were similar, the one with the highest frequency was chosen to represent the specific anxiety level. This indicates that the new VFAS has the property of rank order. By narrowing down the number of facial expressions from 11 to 6, the scaled items are fairly equally spaced and not overlapping, and this support for the application of the VFAS as an interval scale measure.

This was a preliminary study designed to validate a new instrument for assessing anxiety which utilized experienced perioperative healthcare workers rather than actual surgical patients. The clinical relevance is limited because we utilized healthcare professionals as ‘surrogate’ patients in this preliminary evaluation of the new six-facial VFAS. When healthcare professionals utilized this instrument for assessing the level of acute anxiety, it displayed evidence of interval scale properties of rank order and equality between the points on the scale. The healthcare provider can very quickly and easily ask the patient to choose a facial expression which corresponds to their current level of anxiety.

One of the major limitations of this preliminary study is absence of clinically-anxious study population. Only healthcare providers (namely, staff anesthesiologists, anesthesiology residents and perioperative nurses) were invited to participate in this pilot study. However, since all of these individuals work in the preoperative area, they are familiar with evaluating perioperative anxiety in patients. Further studies are clearly needed to validate this new facial anxiety scale in a surgical patient population with varying levels of preoperative anxiety. These preliminary data demonstrate the potential clinical utility of the proposed new VFAS for assessing preoperative [state] anxiety.

### Conclusions

The new six-facial VFAS appears to be a valid tool for assessing the severity of acute [state] anxiety, and could be easily implemented in routine clinical practice without adding significant additional work for the clinical staff providing caring to surgical patients.
